# Health literacy and health promoting behaviors among inpatient women during COVID-19 pandemic

**DOI:** 10.1186/s12905-022-01652-x

**Published:** 2022-03-17

**Authors:** Ali Reza Yusefi, Eshagh Barfar, Salman Daneshi, Mohsen Bayati, Gholamhossein Mehralian, Peivand Bastani

**Affiliations:** 1 Department of Public Health, School of Health, Jiroft University of Medical Sciences, Jiroft, Iran; 2 Health Promotion Research Center, Zahedan University of Medical Sciences, Zahedan, Iran; 3 Department of Public Health, Jiroft University of Medical Sciences, Jiroft, Iran; 4 Present Address: Health Human Resources Research Center, School of Health Management and Information Sciences, Shiraz University of Medical Sciences, Shiraz, Iran; 5 School of Pharmacy, Shahid Beheshti University of Medical Sciences, Tehran, Iran; 6 Faculty of Health and Behavioral Sciences, School of Dentistry, University of Queensland, Brisbane, QLD 4072 Australia

**Keywords:** Health literacy, Health-promoting behaviors, Women, Hospital, COVID-19

## Abstract

**Background:**

One of the leading health indicators during the COVID-19 crisis is health literacy and health-promoting behaviors. The present study aimed to investigate health literacy and health-promoting behaviors among women hospitalized during the COVID-19 pandemic in the southern part of Iran in 2020.

**Methods:**

This descriptive-analytical study encompassed 465 women hospitalized and treated in none teaching hospitals affiliated with the Shiraz University of Medical Sciences. Data collection tools were the Health Literacy for Iranian Adults (HELIA) and Health Promoting Lifestyle Profile II (HPLP-II). The collected data were analyzed using descriptive and inferential statistical methods.

**Results:**

The mean scores of the participants’ "health literacy" and "health-promoting behaviors" were 64.41 ± 11.31 and 112.23 ± 16.09, respectively, indicating the poor level of health literacy and the average level of health-promoting behaviors. Moreover, there was a significant direct correlation between health literacy and health-promoting behaviors (*P* < 0.001, r = 0.471). Furthermore, all health literacy dimensions of comprehension (*P* < 0.001), accessibility (*P* < 0.001), reading skills (*P* < 0.001), evaluation (*P* = 0.002), and decision making and behavior (*P* = 0.003) were detected as the predictors of health-promoting behaviors. Further, statistically significant relationships were noticed between the mean score of health literacy with age (r =  − 0.327, *P* = 0.007), level of education (F = 3.119, *P* = 0.002), and place of residence (t = 2.416, *P* = 0.004) and between health-promoting behaviors with level of education (F = 3.341, *P* = 0.001) and marital status (F = 2.868, *P* = 0.02).

**Conclusion:**

According to the findings, health policymakers should adopt national measures for educational planning to promote health literacy and support health-promoting behaviors to encourage women to adopt a healthy lifestyle.

## Introduction

In late December 2019, a new strain of coronavirus, called COVID-19, was reported from Wuhan, China, which aroused high levels of anxiety and panic worldwide since the disease spread rapidly to other parts of the world [1]. The high transmission rate of the virus and the lack of a specific treatment brought countries large numbers of infected individuals and posed challenges in various health, economic, political, and social areas [2]. Since the onset of the COVID-19 pandemic, vast amounts of information, true or false, confirmed or not, has been provided via official and unofficial sources, which has aroused fear and concern among individuals [3]. Under this condition, in the absence of appropriate health literacy, individuals fail to distinguish true and false information to prevent the disease and pay attention to fake news and unreliable information. This issue would affect their health behaviors [4]. Accordingly, under the critical condition of the COVID-19 pandemic, sufficient health literacy is an essential tool for self-care and the prevention of this disease [5].

According to the World Health Organization (WHO), health literacy refers to cognitive and social skills determining individuals’ motivation and ability to access, understand, and use the information to maintain and promote their health [6]. Although the WHO has introduced health literacy as one of the leading health determinants [7], some studies have indicated the low level of this factor in different communities. Tehrani Bani Hashemi et al. (2007) examined health literacy and factors affecting this variable in 15 provinces of Iran and found out that health literacy in Iran is low [8]. Moreover, a study on health literacy levels in some European countries also revealed that more than half of adults have no sufficient health literacy and no ability and competence to take care of themselves [9]. The low levels of health literacy are detrimental to both individuals and society as a whole [10]. Diviani et al. [11] investigated the relationship between health literacy and information evaluation skills and concluded that individuals with low levels of health literacy have less ability to evaluate information, understand quality, and trust information.

Health literacy is a prominent skill for making appropriate decisions about health behaviors [12], the low level of such literacy can lead to delays in the early diagnosis of diseases, inability to self-care skills, increased use of emergency services, the enhanced incidence of diseases, and increased mortality rate in individuals [13]. Furthermore, patients with low health literacy are hospitalized more frequently and have more extended hospital stays than patients with sufficient health literacy [14]. Lack of health literacy is also correlated with the provision of poor health care and imposes an additional financial burden on health systems [14].

On the other hand, health-promoting behaviors are another health determinant, which plays a critical role in disease prevention [15]. These behaviors include behaviors affecting individuals’ health and behaviors such as seeking health information, performing general examinations, having physical activities, being on an appropriate diet, having proper sleep quality, establishing healthy relationships, and becoming sensitive to diseases [16]. These behaviors are of paramount importance since they prevent diseases, reduce pathogenicity, improve the quality of life, and decrease the burden of health care in societies [17]. These behaviors have been examined in Iran and other countries among different groups of adolescents [18, 19], professors [20, 21], students [22], workers and employees [23, 24], and women [25–27], according to which different findings have been reported. More specifically, Lee et al. evaluated health-promoting behaviors and showed the average level of these behaviors in women [28]. Furthermore, Mohammadalizadeh et al. [29] reported that Iranian women exhibited moderate levels of health-promoting behaviors and performed well in the subscale of social relations; however, their lowest scores were in the physical activity subscale.

Although health literacy and health-promoting behaviors are of significance to all members of society, the issue is of paramount importance to some population groups, including women. Since training is critical to promoting child and family health, women have been identified as the primary population in need of increased literacy and health behaviors [30]. Culturally, women play a key role in bringing and establishing health conditions in almost all societies [31]; to fulfill this role, they should maintain and promote their health status properly [32]. In this line, health literacy significantly helps women involve effectively in all health activities related to themselves and their surroundings [33, 34]. Otherwise, women rarely make a right decision regarding their health conditions and how purse such a crucial thing in their life, leading to have unhealthy families or at a large scale an unhealthy community [35]. Given these logics, the authors believe that women would be one the best group of populations to study their health literacy and subsequent health-promoting behaviors. On the other hand, in patients admitted to medical centers, an adequate level of health literacy and appropriate health behaviors play an effective and significant role in accelerating the recovery process and following health care instructions, thereby accelerating patients' recovery [36–40]. All in all, this study aimed to investigating the health literacy and health-promoting behaviors among women hospitalized in public hospitals during the COVID-19 pandemic.

## Methods

### Design and setting

This descriptive-analytical cross-sectional study was conducted in 2020.

### Participants

The study population encompassed all women admitted to nine teaching hospitals affiliated to the Shiraz University of Medical Sciences in Shiraz (namely Namazi, Shahid Dastgheyb, Shahid Chamran, Khalili, Hafez, Shahid Faghihi, Ibn Sina, Hazrat Zeinab, and Shahid Rajaee hospitals). The women were hospitalized at the time of the study and were receiving care services. Considering 95% confidence level and the correlation between health literacy and health-promoting behaviors, the sample size was estimated to be at least 386 persons in previous studies. In this study, 465 persons were included to increase accuracy and prevent bias resulting from sample loss. Regarding the sample size, the stratified sampling method was used to select the participants. To this end, the researcher first identified the total number of women admitted to different wards by referring to each hospital. Then the hospital with more female patients had a larger sample size, proportionate to its size. Moreover, in each hospital, patients were selected and evaluated in proportion to the number of women hospitalized in each ward (the ward with the largest number of hospitalized women in proportion to the volume encompassed a larger portion of the sample size). Inclusion criteria were informed consent, the ability to speak and read and write, and no cognitive problems. No interest in participation, lack of enough time, and incomplete filling of the survey were the among the exclusion criteria we have considered in this study.

### Instruments

Data collection tools included a three-section standard questionnaire. The first section addressed demographic information (namely age, place of residence, marital status, level of education, and monthly income), and the second and third sections dealt with the standard Health Literacy for Iranian Adult (HELIA) and Health -Promoting Lifestyle Profile II: (HPLP-ll).

The HELIA Questionnaire is a 33-item scale and evaluates individuals’ ability in different dimensions of health literacy, including reading skills (4 items), accessibility (6 items), comprehension (7 items), evaluation (4 items), and decision making and behavior (12 items). The questionnaire is scored based on a 5-point Likert scale. The scoring is as follows in the items on reading skills: 5 = quite easy, 4 = easy, 3 = neither easy nor difficult, 2 = difficult, and 1 = quite difficult. Regarding the other four dimensions, the scoring is as follows: 5 = always, 4 = often, 3 = sometimes, 2 = rarely, and 1 = never. To score this tool, the raw score of each individual from each of the subscales is obtained from the sum of scores. Then to convert this score to a 0–100 range, the "difference in gross score obtained from the minimum probable raw score" is divided by the "difference between the maximum probable score and the minimum probable score." Finally, the scores of all subscales (based on a 0–100 range) are added and divided by the number of subscales [5] to calculate the total score. In this regard, scores 0–50, 50.1–66, 66.1–84, and 84.1–100 are considered inadequate, poor, adequate, and excellent health literacy, respectively [30]. The validity and reliability of this questionnaire were confirmed in Montazeri et al. study [30]. They reported Cronbach's alpha equal to 0.89. Further, the face and content validity of the questionnaire have been widely investigated by wider researchers in areas such as healthcare management, social medicine, mental health, and so on [30].

The HPLP-ll consists of 52 items addressing six dimensions: Nine items on weight control and nutrition (score range 9–36), eight items on physical activity (score range 8–32), eight items on stress management (score range 8–32), nine items on spiritual growth (score range 9–36), nine items on interpersonal relationships (score range 9–36), and nine items on responsibility towards health (score range 9–36). All items were scored based on a Likert scale ranging from one to four (1 = never, 2 = sometimes, 3 = often, 4 = always). The total score range of this questionnaire (i.e., the sum of scores in the above six dimensions) ranges from 52 to 208: poor = 52–91, average = 92–130, good = 131–169, and excellent = 170–208. Previous research not only evaluated the validity of HPLP-ll but also reported generally agreed estimations of Cronbach's alpha for HPLP-ll [41–43].

### Procedure and statistical analysis

Data were collected by completing questionnaires and face-to-face interviews held by the researcher. To this end, the researcher referred to the wards where the women were hospitalized. After providing the necessary explanations about the research objectives, while ensuring the women regarding the confidentiality of their personal information, the researcher asked the women to participate in the study if they were willing and answer the researcher's questions carefully. After completing the questionnaires, the data were imported to the SPSS software version 23 for descriptive and inferential statistical methods. We performed Pearson’s correlation to test the relationship between health literacy and health-promoting behaviors and the relationship between the main variables of research with age. t-test has been used to investigate the mean difference between the main variables of the research with the place of residence. To analyze if there are any difference between the main variables of research and participants profile such as marital status, education, and income level variables, ANOVA test has been applied. Further, we performed multiple linear regression to determine the simultaneous effect of different dimensions of health literacy on health-promoting behaviors.

## Results

According to Table 1, most of the women were in the age range of 20–35 years (39.14%), urban residents (58.71%), married (81.09%), with a diploma and higher education (68.60%) and income levels ranging from ten to twenty million Rials (52.90%).

**Table 1:** Frequency distribution of the participants’ demographic information

Variable	Category	Frequency	Percentage
Age (years)	< 20	59	12.69
	20–35	182	39.14
	36–50	169	36.34
	> 50	55	11.83
Total	–	465	100
Place of Residence	Rural areas	192	41.29
	Urban areas	273	58.71
Total	–	465	100
Marital Status	Single	53	11.39
	Married	377	81.09
	Divorced	26	5.59
	Widowed	9	1.93
Total	–	465	100
Education Level	Illiterate	23	4.95
	Primary school	51	10.97
	Middle school	72	15.48
	Diploma and higher	319	68.60
Total	–	465	100
Income level (per month)	No income	167	35.92
	10–20 million Rials	246	52.90
	21–30 million Rials	46	9.89
	+ 30 million Rials	6	1.29
Total	–	465	100

The mean scores of the participants’ "health literacy" and "health-promoting behaviors" were 64.41 ± 11.31 and 112.23 ± 16.09, respectively, indicating the poor level of health literacy and the average level of health-promoting behaviors. As we can see in Table 2, among the health literacy dimensions, the highest and lowest mean scores were obtained for the reading skills dimension (66.49 ± 12.49) and the evaluation dimension (62.27 ± 11.42), respectively. Moreover, among the dimensions of health-promoting behaviors, the highest and lowest mean scores were obtained for spiritual growth (22.12 ± 12.64) and physical activity (12.43 ± 6.26), respectively (Table 2).

**Table 2:** The mean and standard deviation of health literacy and health-promoting behaviors among the participants in 2020

Variables	Dimensions	Mean	SD
Health literacy	Accessibility	65.39	11.89
	Reading skills	66.49	12.49
	Comprehension	64.51	12.42
	Evaluation	62.27	11.42
	Decision making and behavior	63.36	11.28
	Total health literacy	64.41*	11.31
Health-promoting behaviors	Spiritual growth	22.12	12.64
	Interpersonal relationships	20.16	9.11
	Weight control and nutrition	19.56	7.21
	Responsibility towards health	18.27	10.21
	Stress management	19.69	8.14
	Physical activity	12.43	6.26
	Total health-promoting behaviors	112.23**	16.09

^*^Score out of 100

^*^Score out of 208

According to Table 3, there is a positive and significant correlation between health literacy and its dimensions with health-promoting behaviors (r = 0.471 and *P* < 0.001). Furthermore, among the health literacy dimensions, comprehension had the strongest correlation with health-promoting behaviors (r = 0.501 and *P* < 0.001).

**Table 3:** Relationship between health literacy and health-promoting behaviors among the participants in 2020

		Health literacy					
	Dimensions	Accessibility	Reading skills	Comprehension	Evaluation	Decision making and behavior	Total health literacy
Health-promoting behaviors	Spiritual growth	r* = 0.214 P** = 0.01	r = 0.261 *P* = 0.01	r = 0.201 *P* = 0.02	r = 0.196 *P* = 0.03	r = 0.188 *P* = 0.04	r = 0.213 *P* = 0.03
	Interpersonal relationships	r = 0.361 *P* = 0.002	r = 0.349 *P* = 0.003	r = 0.427 *P* = 0.004	r = 0.372 *P* = 0.001	r = 0.367 *P* = 0.002	r = 0.378 *P* = 0.002
	Weight control and nutrition	r = 0.523 *P* = 0.001	r = 0.536 *P* = 0.001	r = 0.573 *P* < 0.001	r = 0.556 *P* < 0.001	r = 0.511 *P* = 0.001	r = 0.541 *P* < 0.001
	Responsibility towards health	r = 0.532 *P* < 0.001	r = 0.513 *P* = 0.002	r = 0.588 *P* < 0.001	r = 0.526 *P* = 0.001	r = 0.537 *P* < 0.001	r = 0.545 *P* < 0.001
	Stress management	r = 0.451 *P* = 0.001	r = 0.462 *P* = 0.001	r = 0.489 *P* < 0.001	r = 0.443 *P* = 0.002	r = 0.473 *P* = 0.001	r = 0.466 *P* = 0.001
	Physical activity	r = 0.607 *P* = 0.002	r = 0.611 *P* = 0.001	r = 0.661 *P* < 0.001	r = 0.622 *P* = 0.001	r = 0.631 *P* < 0.001	r = 0.631 *P* < 0.001
	Total health-promoting behaviors	r = 0.476 *P* < 0.001	r = 0.466 *P* < 0.001	r = 0.501 *P* < 0.001	r = 0.461 *P* = 0.001	r = 0.454 *P* = 0.002	r = 0.471 *P* < 0.001

^*^r Pearson Correlation Coefficient

^**^P P-Value, Correlation is significant at the 0.05 level

Regarding the effect of different dimensions of health literacy on the participants’ health-promoting behaviors, Table 4 revealed that the significant variables in the model, which were determined using the Enter method, were the significance of comprehension, accessibility, reading skills, evaluation, and decision making and behavior, respectively. Further, the coefficient of determination for the processed model (R-Adjusted) of this test was 0.58, suggesting that 58% of the variations in the scores of health-promoting behaviors can be explained by the model variables. According to the multiple linear regression analysis of the linear equation, the scores of health-promoting behaviors were obtained as follows:

$${\text{Y}} = 0.501 + 0.321{\text{x}}_{1} + 0.287{\text{x}}_{2} + 0.257{\text{x}}_{3} + 0.221{\text{x}}_{4} + 0.194{\text{x}}_{5}$$

where *Y* is the score of health-promoting behaviors, and *x* is the various dimensions of health literacy.

**Table 4:** Variables affecting participants’ health-promoting behaviors, determined

Variable definition	Variable	Unstandardized coefficients		Standardized coefficient β	t	*P*-value *
		B	SE			
–	(Constant)	0.501	1.07	–	2.98	0.001
x_1_	Comprehension	0.321	0.059	0.352	2.81	< 0.001
x_2_	Accessibility	0.287	0.068	0.309	2.74	< 0.001
x_3_	Reading skills	0.257	0.071	0.279	2.66	< 0.001
x_4_	Evaluation	0.221	0.092	0.243	2.24	0.002
x_5_	Decision making and behavior	0.194	0.078	0.216	1.94	0.003

^*^P-value Correlation is significant at the 0.05 level, *B* Unstandardized coefficients, *SE* standard error

The findings depicted in Table 5 indicated a statistically significant relationship between the mean score of the participants’ health literacy with age (r =  − 0.327, *P* = 0.007), level of education (F = 3.119, *P* = 0.002) and place of residence (t = 2.416, *P* = 0.004). Accordingly, in the age group above 30 years, “poor health literacy" increased significantly. Moreover, health literacy was higher in women living in urban areas than those residing in rural areas and women with higher levels of education compared to those with lower levels of education. Further, a statistically significant relationship was observed between health-promoting behaviors with education level (F = 3.341, *P* = 0.001) and marital status (F = 2.868, *P* = 0.02). Accordingly, the mean score of health-promoting behaviors was higher in women with a diploma or higher degrees and the married.

**Table 5:** Relationship between health literacy and health-promoting behaviors with the participants’ demographic information

Demographic information	Variables	Type of test and significant*	
		(r_p_)*	*P* value
Age	Health literacy	− 0.327	**0.007**
	Health-promoting behaviors	0.143	0.11

		(T)*	*P* value
Place of residence	Health literacy	2.416	**0.004**
	Health-promoting behaviors	1.634	0.09

		(F)*	*P* value
Marital status	Health literacy	2.132	0.07
	Health-promoting behaviors	2.868	**0.02**
Education level	Health literacy	3.119	**0.002**
	Health-promoting behaviors	3.341	**0.001**
Income level	Health literacy	2.301	0.06
	Health-promoting behaviors	2.086	0.08

^*^r pearson correlation coefficient, **t** T-Test, **F** Test ANOVA, (Bold indicates that correlation is significant at the 0.05 level)

## Discussion

Given the significant role of health literacy and health-promoting behaviors in individuals’ health and their relationship, the present study aimed to investigate health literacy and health-promoting behaviors among women hospitalized in nine teaching hospitals affiliated with the Shiraz University of Medical Sciences during the COVID-19 pandemic.

The study findings indicated the participants’ poor health literacy level. Similarly, the findings of Panahi et al. [44], Azimi et al. [6], Sharif-Moghadam et al. [45], Nekoei-Moghadam et al. [46], Paasche-Orlow et al. [47], and Mahmoudi and Taheri [48] revealed the borderline and average health literacy levels among their participants. In Ghaffari et al. study, which was to investigate the health literacy of women referring to health centers in Zanjan, the findings suggested that 22.6% of the subjects had inadequate health literacy, and that 22.3% of the participants had borderline health literacy [49]. Furthermore, the findings of Ghanbari et al. study on the health literacy of pregnant women referring to the health centers affiliated to the Shahid Beheshti University of Medical Sciences in Tehran indicated that 30% of the women had inadequate health literacy and that 24.6% of the participants had borderline health literacy [36]. In Afshari et al. study, in line with the present findings, 71.9% of the participants had inadequate and borderline literacy [50]. Health literacy is considered as a vital and remarkable indicator in the health care outcomes and costs and is a prerequisite in today’s health care systems. Health literacy in health care recipients can be regarded as a critical factor affecting individuals’ decisions and how they act to promote and improve their health, especially during the COVID-19 pandemic [51]. In other words, improving health literacy skills by improving understanding, comprehension, and evaluating the benefits of preventive and diagnostic behaviors can be one of the main factors in preventing and managing COVID-19 disease [44]. McCaffery et al. claim that individuals with inadequate health literacy have less understanding of COVID-19 symptoms, less detect anti-infective behaviors, and have more difficulty finding information and understanding government messages about COVID-19 than those with those who are concerned with adequate health literacy. Individuals with inadequate health literacy consider the social distance a critical issue and have more difficulty remembering and accessing medication during lock-downs. The researchers also conclude that individuals with inadequate health literacy are more likely to confirm misconceptions about COVID-19 and vaccination than those with adequate health literacy [52].

The findings suggested that women’s health-promoting behaviors are at a "moderate" level, and this finding is in line with those of other similar studies. In a study in Saudi Arabia, Ashgar et al. evaluated the effect of the COVID-19 pandemic on adult’s health-promoting behaviors in Jazan. They found out that, on average, participants "sometimes" exhibited health-promoting behaviors [53]. Among the dimensions of health-promoting behaviors, spiritual growth had the highest mean score in the present study. This is consistent with the findings of Mirjalili et al. [54], Gokyildiz et al. [55], and Enjezab et al. [56]. The high mean score of spiritual growth among the women may be caused by the Islamic culture of the Iranian people because religion is a key factor in the growth and regulation of relationships among individuals in society [57].

On the other hand, the physical activity dimension had the lowest mean score compared to other dimensions. This finding is in agreement with the findings of Najafabadi and Rezaei [58] and Kalroozi et al. [59]. The findings of a national survey of Iranian adults suggested that above 80% of the Iranian population is physically inactive [60]. In this regard, inactivity is a health problem of a cultural and social origin. Participation in physical and sports activities depends on various factors such as perception of physical activity, self-confidence, social support, and adequate motivation [61]. In their study, Heidari and Kermanshahi introduced excessive workload, lack of time, and being busy because of the requirements of family life as the primary individual barriers to the lack of physical activity and regular exercise among individuals [62].

On the other hand, such inactivity and lack of regular physical activity seem to be rooted in the wrong lifestyle and rapid development of civilization and industrialization [63]. Regular and planned physical activities can improve and enhance women's health and prevent diseases and major disorders [63]. According to many studies, physical activity positively affects menopausal symptoms and prevents osteoarthritis, osteoporosis, cardiovascular diseases, hypertension, diabetes, and obesity [56, 64]. In this regard, the most remarkable barriers to having regular exercises are lack of awareness, lack of motivation, and lack of social support, weather conditions, lack of companions to participate in sports activities, lack of interest, lack of time, family commitments, and lack of access to gyms [65, 66]. Undoubtedly, the COVID-19 pandemic will be the basis of many health-related developments in individuals’ lives, according to which people's lifestyles are predicted to change, and many healthy habits and behaviors are gradually institutionalized in society [67].

Like many other similar studies [7, 19, 68–72], the present findings showed a positive and significant correlation between health literacy and its dimensions with the health-promoting behaviors, indicating the main and significant function of health literacy in promoting individuals’ health status. According to this finding, improving health literacy in women can lead to a wide range of improvements in their health status, including physical, mental, and spiritual health. In other words, women with higher health literacy select healthier behaviors leading to their improved health status. Evidence suggests the relationship between low health literacy with increasing hospitalization rates, emergency services, difficulty taking medications and understanding health messages, having poorer general health status and higher mortality rates, and decreasing preventive services such as screening and vaccines [73]. Halverson et al. [74] also consider inadequate health literacy as a barrier to the physician–patient relationship as individuals with inadequate health literacy cannot judge, select, and analyze the information presented by health care providers to make decisions to improve their health.

The Pearson’s correlation test results revealed a statistically significant relationship between health literacy and the participants’ demographic information, including age, level of education, and place of residence. In the present study, the poor level of health literacy increased significantly with increasing age among women aged 30 years. This finding is consistent with those of other similar studies [73, 75–77]. It seems that with aging, there is inadequacies in individuals’ literacy, which is the result of reduced cognitive function, distance from years of formal education, and reduced sensory abilities [78].

Moreover, the significant relationship between health literacy and age has been reported in many studies, including Ghanbari et al. [36], Lee [51], Cho [79], McLaghlin [80], Hironaka [81], and Paasche-Orlow et al. [47]. Similarly, the findings of Mahmoudi and Taheri [48] and Ghanbari et al. [36] also indicated a significant relationship between health literacy and level of education. This relationship has been observed in many other studies, including case, review, and national studies [49, 75, 76, 82, 83]. The findings of a systematic review study on health literacy by the Agency for Healthcare Research & Quality (AHRQ) in 2012 reported the low levels of health literacy as a significant problem in the United States. This comes especially true for individuals with education levels below the high school diploma. According to this report, the years and level of education are strong predictors of health literacy. In the US National Health Literacy Survey, above three-fourths of the participants who had not graduated from high school had "lower-basic" or "basic" health literacy; however, the rate was 13 percent in individuals with a four-year college education [75]. In their study, Sanders et al. concluded that 66% of individuals with limited health literacy held diplomas [84]. Increasing levels of education can play a facilitating role in increasing health literacy; hence, increased awareness leads to variations in attitudes and promotes understanding and comprehension of various aspects of personal health.

The other finding of the present study suggested a statistically significant relationship between health literacy and place of residence as such the level of health literacy is higher among women living in urban areas than those in rural areas. In this regard, the higher levels of health literacy among urban women, compared to rural women, can be justified and explained based on the urban residents’ greater access to educational, equipment, and communication facilities.

The Pearson’s correlation test results also showed a statistically significant relationship between women's health-promoting behaviors with the level of education and marital status. According to the present study's findings, women with a diploma or higher education exhibit more health-promoting behaviors than women with lower levels of education. This finding is in line with those of studies conducted by Mirjalili et al. [54], Anbari et al. [85], and Gokyildiz [55]. The higher level of education among women and consequently the possibility of their further participation in society and receiving social support are factors affecting health-promoting behaviors. This is while women with lower levels of education are not fully aware of health threats and are less likely to follow health-promoting behaviors. The study findings suggested that married women exhibited higher levels of health-promoting behaviors than single women. This implies that the presence of a spouse increases the likelihood for women to follow up health-promoting behaviors. Kalroozi et al. [59] and Moghadam Tabrizi et al. [86] claimed that marital status was a critical factor in exhibiting health-promoting behaviors.

Green and Rodgers also suggest that married individuals are happier by controlling a variety of factors, exhibit higher levels of health-promoting behaviors, enjoy higher levels of physical and mental health, and have a longer life expectancy [87]. Evidence shows that married people benefit more from the social and economic factors affecting health status [88].

One of the limitations of the present study was the participants’ physical condition and gender. The researchers spared their efforts to remove such a limitation by clarifying the objective of the study and referring to the participants when they were in better physical conditions.

## Conclusion

The study findings revealed the poor levels of health literacy and the moderate level of health-promoting behaviors among women admitted to the teaching hospitals affiliated with the Shiraz University of Medical Sciences during the COVID-19 pandemic. Accordingly, health policymakers should adopt national measures for educational planning to improve health literacy and support health-promoting behaviors to encourage women to adopt a healthy lifestyle. Social media may be considered as an efficient means to increase health literacy and subsequent health-promoting behaviors of women.

Given that limited and poor health literacy prevents the proper understanding of health messages and recommendations, hospital staff is recommended to effectively transmit information to such hospitalized women. It is also suggested to implement health education programs with an emphasis on the development of expert sports plans and physical activity to improve health-promoting behaviors. It is recommended that governments should provide their comprehensive support of pushing women toward health-promoting behaviors.

Future studies are also suggested to detect the most cost-effective interventions to improve women's health literacy and adopt health-promoting behaviors on a national scale. The findings of this study will inform policy maker to set priorities they need to improve the health literacy of women and help them as to how to promote their health-promoting behaviors.

## Data Availability

The datasets generated and analyzed during the current study are not publicly available due to confidentiality data but are available from the corresponding author on reasonable request.
